# Intra and inter variation in training load, recovery state and technical–tactical performance across a standard microcycle in Sub-elite youth football players

**DOI:** 10.3389/fspor.2025.1720353

**Published:** 2026-01-12

**Authors:** Pedro Afonso, Pedro Forte, Luís Branquinho, Ricardo Ferraz, Nuno Domingos Garrido, José Eduardo Teixeira

**Affiliations:** 1 Biosciences Higher School of Elvas, Polytechnic Institute of Portalegre, Portalegre, Portugal; 2 Department of Sport, Exercise and Health Sciences, University of Trás-os-Montes e Alto Douro, Vila Real, Portugal; 3 Research Centre for Active Living and Wellbeing (Livewell), Instituto Politécnico de Bragança, Bragança, Portugal; 4 Department of Sports, Higher Institute of Educational Sciences of the Douro, Penafiel, Portugal; 5 CI-ISCE, ISCE Douro, Penafiel, Portugal; 6 Department of Sports Sciences, Instituto Politécnico de Bragança, Bragança, Portugal; 7 Life Quality Research Center (LQRC-CIEQV), Santarém, Portugal; 8 Department of Sport Sciences, University of Beira Interior, Covilhã, Portugal; 9 Research Center in Sports Sciences, Health Sciences and Human Development (CIDESD), Vila Real, Portugal; 10 Department of Sports Sciences, Polytechnic Institute of Guarda, Guarda, Portugal; 11 Department of Sports Sciences, Polytechnic of Cávado and Ave, Guimarães, Portugal; 12 SPRINT—Sport Physical Activity and Health Research & Innovation Center, Guarda, Portugal

**Keywords:** youth football, training load, recovery, tactical performance, sub-elite

## Abstract

**Introduction:**

Monitoring youth football requires integrating physical, perceptual, recovery, and tactical dimensions. However, evidence in younger sub-elite cohorts (U11–U13) remains scarce. This study aimed to analyze intra and inter variation in external load, internal load, recovery, and technical–tactical indicators across a competitive microcycle, comparing U11 and U13 sub-elite players. We hypothesized that (i) the match would elicit the highest objective intensities, while training would be perceived as more demanding, and (ii) U13 players would outperform U11 in high-intensity and tactical outcomes, whereas U11 would show higher perceived exertion and greater motor irregularity.

**Methods:**

Forty male sub-elite players (U11 = 30; U13 = 10) were monitored across a competitive microcycle (MD-4 to MD). External load was assessed via GPS (TD, AvS, HSR, HID, sprints, MRS, ACC, DEC), internal load through HR (U13 only) and session-RPE, recovery via TQR, and technical–tactical performance using FUT-SAT (DMI, MEI).

**Results:**

The match elicited the highest intensities in HSR, MRS, AvS, and HID, while all training sessions were perceived as ∼400 AU more demanding in sRPE than MD. U13 players outperformed U11 in intensity-and velocity-based measures (HSR +166%, sprints +150%, MRS +5%), while U11 showed higher TD (+10%), ACC (+23%), DEC (+29%), and sRPE (+6%). HR data in U13 revealed greater Z5 exposure in MD-4 vs. MD-1 and higher Z2 time on MD-1. In the tactical domain, U13 displayed superior offensive coverage effectiveness in both DMI and MEI, with no differences in other principles.

**Discussion:**

These findings demonstrate that the microcycle followed a structured pattern, with matches concentrating objective intensity and training sessions eliciting greater perceived effort. Practically, training for U11 should emphasize motor efficiency and load regulation, while U13 programs should target high-intensity capacity and tactical coordination. Over time, integrating multidimensional monitoring (GPS, sRPE, TQR, FUT-SAT) may guide coaches in aligning training stimuli with long-term development goals, bridging physical, perceptual, and tactical competencies in sub-elite youth football.

## 1 Introduction

Football performance results from the dynamic interaction of physical, technical, tactical, and psychological dimensions, whose integration simultaneously influences performance, recovery, and player well-being (1). Understanding these multidimensional relationships is essential to sustain high levels of performance, reduce the risk of excessive fatigue, and optimize the training process (2, 3). Therefore, monitoring the training load will help to better adequate the training sessions intensity and volume.

Training load (TL) has been conceptualized in different ways. Some authors have proposed a three-dimensional framework—internal, external, and well-being—which has recently been applied in sub-elite youth football (4) and also supported in other sports contexts (5, 6). However, most football research has preferred to adopt a two-dimensional approach, dividing TL into external and internal load (7–10). In sub-elite contexts, this two-dimensional model has been consistently applied (4, 11, 12) although recent studies have also explored the integration of recovery quality and effort perception, thus connecting TL to well-being (13, 14). In this framework, external load reflects the actual mechanical work performed, while internal load represents the physiological (8, 12) and psychophysiological responses of the organism (4, 12, 15, 16). External load can be monitored using GPS-based tracking systems, which quantify variables such as total distance (TD), velocity, accelerations (ACC), and decelerations (DEC), while internal load is commonly assessed via objective indicators, such as HR or lactate, or subjective measures such as Rating of Perceived Exertion (RPE), Session RPE (sRPE) and Total Quality Recovery (TQR) (4, 11).

Among the objective indicators of internal load, HR has received particular attention due to its ability to quantify relative intensity through percentage of maximal heart rate (%HRmax) (8, 17) or the distribution of time spent in predefined intensity zones (18, 19). Previous studies have shown that HR is sensitive to cognitive fatigue, reflecting simultaneous alterations in physical performance and psychophysiological markers (17). In training contexts, HR variations can be predicted from GPS-derived metrics, with the change in heart rate (HR*Δ*) considered a useful marker of adaptation and fitness status (20). The validity of these load measures has been widely confirmed, with strong correlations reported between RPE and HR responses (10, 21), and between sRPE and GPS-derived variables (22). Furthermore, sRPE has been validated as an effective and non-invasive monitoring tool in elite youth players (∼17 years) (10). Additional evidence in youth players (U15–U19; *n* = 456) confirmed that the match constitutes the most demanding stimulus of the microcycle, with a pronounced load reduction on MD-1, illustrating the tapering pattern (23). Consistently, in sub-elite U15–U19 players, external load has been shown to increase with age (4). However, in younger players, the reliability of perceptual measures such as RPE must be carefully considered, as their accuracy depends on the child's ability to understand and consistently apply rating scales. Previous studies have shown that, when adapted to age-appropriate formats and preceded by familiarization sessions, children are capable of reliably self-reporting RPE values (4, 11, 24, 25). This ability appears to hold even in performance-oriented training sessions with high heart rate-based internal load values, although some limitations may emerge in such contexts (26). Nevertheless, many protocols still recommend that the coach or researcher provides verbal clarification to ensure consistency and comprehension.

The monitoring of recovery and well-being is a key component of training and competition management (1). Among the available subjective tools, the Total Quality Recovery (TQR) scale is widely used to assess global recovery perception (27). Its utility has been consistently demonstrated: in U18 players, TQR was sensitive to match-induced fatigue and predictive of subsequent physical performance, particularly in accelerations and decelerations (23); in elite female players, recovery scores remained relatively stable throughout the microcycle despite fluctuations in external load (28); and longitudinal evidence in sub-elite youth (U15–U19) indicated age-related differences in training load and recovery status, with older players (U17/U19) showing greater tolerance to higher loads, whereas younger players (U15) exhibited greater variability in recovery measures (11). However, no study has yet examined recovery and well-being in younger sub-elite cohorts such as U11 and U13, highlighting a critical gap in the literature.

Beyond load and recovery, the technical–tactical dimension is also decisive for football performance (29). Declarative tactical knowledge (knowing what to do) and procedural knowledge (knowing how to act) underpin the effectiveness of both individual and collective actions (30). Several instruments have been proposed to assess these dimensions, such as the Team Sports Performance Assessment Procedure (TSAP) (31), the Game Performance Assessment Instrument (GPAI) (32), and, specifically for football, the Football Tactical Assessment System (FUT-SAT) (33). FUT-SAT enables the analysis of offensive principles (penetration, coverage, mobility, space) and defensive principles (delay, coverage, balance, concentration) in small-sided games, and has been widely recognized for its applicability in youth football (34). Importantly, it quantifies performance through composite indexes, namely the Decision-Making Index (DMI) and the Motor Effectiveness Index (MEI). The DMI reflects the proportion of correct decisions relative to total tactical actions, whereas the MEI represents the proportion of effective executions relative to total attempts (33, 35). Together, these indexes provide an integrated measure of tactical awareness and technical execution, offering a comprehensive perspective on player performance, particularly valuable in developmental stages such as U11 and U13.

These findings highlight the need to integrate technical–tactical indexes such as DMI and MEI alongside physical and physiological metrics, particularly in developmental stages where decision-making and motor efficiency are still consolidating (35, 36). More specifically, the standard microcycle in sub-elite football players has already been studied in the under-15 to under-19 age groups, but no research has yet been conducted in the age groups that precede the initiation of the developmental stages with the introduction to football performance (12, 37). Similarly, there is also a lack of understanding of how the perception of recovery states in U11 and U13 football players (13, 38).

Despite these advances, the literature remains scarce in younger age groups, particularly U11 and U13, and especially in sub-elite contexts. Most studies have focused on U15–U19 or elite/professional populations, leaving a gap in understanding how external and internal loads, recovery indicators, and technical–tactical performance interact in early developmental stages. Therefore, integrating these dimensions in younger cohorts is crucial to understanding how physical and tactical learning co-evolve during the early stages of specialization. This multidimensional perspective helps explain how young players adapt to training stimuli, regulate effort and recovery, and translate these processes into effective tactical behavior, essential mechanisms for long-term skill acquisition and injury prevention.

In this context, the term standard microcycle refers to the structured weekly schedule implemented within the academy setting, comprising four consecutive training sessions followed by an official match. Although this structure may differ from elite or professional environments, it represents the consistent, planned format through which sub-elite youth players experience and adapt to football training. Within this framework, analyzing both intra and inter variation provides valuable insights into how players respond to training stimuli. Intra variation reflects each player's capacity to adapt across sessions within a microcycle, capturing day-to-day fluctuations in load, recovery, and performance. In contrast, inter variation highlights developmental heterogeneity between players or age groups, offering a window into how maturation, experience, and training exposure shape physical and tactical behaviors. Together, these perspectives are critical for understanding training adaptation and for guiding individualized planning in developmental contexts (39).

Accordingly, the present study aimed to provide an integrated analysis by (i) examining intra variation in external load, internal load, and recovery across a competitive microcycle and (ii) identifying inter-individual differences between U11 and U13 players, including technical–tactical indicators assessed through the FUT-SAT. We hypothesized that (i) the match would elicit the highest intensities in objective external-load metrics, while training sessions would be perceived as more demanding in terms of psychophysiological cost; and (ii) U13 players would present superior performance in high-intensity physical variables and tactical indexes (DMI and MEI), whereas U11 players would report higher subjective exertion and display greater variability in motor behaviours.

## 2 Materials and methods

### 2.1 Study design

**Figure 1:**
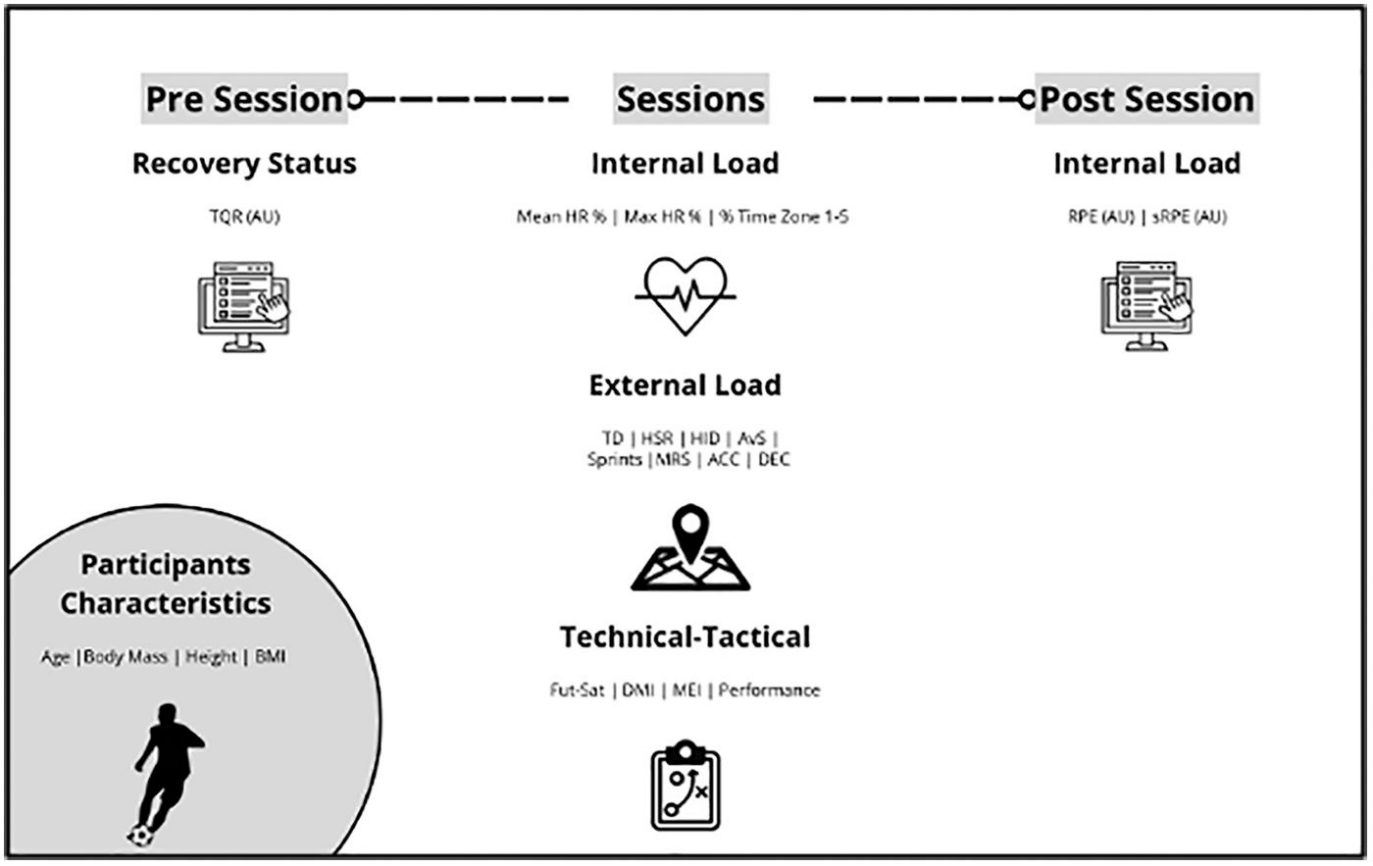
Schematic representation of the study design and variables assessed, illustrating the competitive microcycle structure (MD-4 to MD), showing the temporal sequence of training sessions, recovery and performance assessments. Pre-session assessments included TQR, while post-session measures comprised sRPE. External load and internal load were collected across all sessions.

This study followed an observational design, monitoring a standard competitive microcycle in sub-elite youth football players during the 2024/2025 season. Figure 1 provides a schematic representation of the study design and variables assessed. Before each training session, recovery status was evaluated using the TQR scale. During training sessions and the official match, external load (total distance, high-speed running, high-intensity distance, sprints, maximum running speed, accelerations, decelerations) and internal load (heart rate, sRPE) were assessed. In addition, technical–tactical performance was evaluated during training sessions using the FUT-SAT protocol, which provided indexes of decision-making, motor efficiency, and overall performance. Post-session, additional internal load measures were collected via RPE and sRPE. Anthropometric characteristics (age, body mass, height, BMI) were also recorded at baseline. Detailed procedures for each measure are described in the subsequent subsections.

### 2.2 Participants

Forty male sub-elite football players (U11 = 30; U13 = 10) from the same development academy participated voluntarily in this study. All players were part of the academy's competitive squads for their respective age groups and trained four times per week plus one official match, consistent with the sub-elite standard of structured, year-round participation below national-level academies. Players were recruited directly through the academy's technical coordination staff. Table 1 presents the total sample, including mean and standard deviation for age, height, body mass, and BMI. Anthropometric assessments were conducted using a digital scale (Tanita BC-545N®) and a digital stadiometer (SECA® 242) (40). All participants belonged to the same Portuguese sub-elite academy and were regularly engaged in official training sessions and official competitions.

**Table 1:** Descriptive characteristics of the participants (mean ± SD).

Variable	U11 (*n* = 30)	U13 (*n* = 10)	Overall (*n* = 40)
Age (years)	9.67 ± 0.99	12.10 ± 0.74	10.28 ± 1.41
Body mass (kg)	32.80 ± 8.80	39.80 ± 5.20	34.60 ± 8.60
Height (cm)	140.20 ± 10.20	152.50 ± 5.50	143.30 ± 10.70
BMI (kg/m²)	16.50 ± 2.80	17.10 ± 2.10	16.70 ± 2.60

The procedures were conducted in accordance with the Declaration of Helsinki for research with human participants. Approval was granted by the scientific board of the University of Trás-os-Montes e Alto Douro (UTAD). Written informed consent was obtained from parents or legal guardians, and assent was provided by all players prior to data collection. The eligibility criteria required participants to: (i) take part in a competitive week with only one official match; (ii) complete all four training sessions and the corresponding match; and (iii) be free from injuries or medical conditions that could prevent full data collection. Exclusion criteria included: (i) absence from any training session; (ii) not being selected or available for the official match; (iii) incomplete or missing data from GPS, heart rate, RPE, or TQR assessments; and (iv) any injury or health limitation occurring during the monitoring period.

### 2.3 Experimental approach

This study monitored a standard competitive microcycle composed of four consecutive training sessions (MD-4; MD-3; MD-2; MD-1) and one official match (MD). A total of eight training sessions (four per age group) and two matches (one per age group) were analysed, corresponding to 200 individual observations.

The training sessions lasted approximately 90 min and were performed on synthetic turf fields with official dimensions (100 × 70 m), between 17:00 and 21:00, under comparable environmental conditions (temperature: 9–12 °C; relative humidity: 76%–87%). Matches lasted ∼60 min and were played on synthetic turf fields of official dimensions for 7-a-side football (U11) and 9-a-side football (U13), between 10:00 and 13:00, under similar conditions (temperature: 8–15 °C; relative humidity: 60%–70%). The microcycle followed the match-day minus format: MD-4 (Tuesday), MD-3 (Wednesday, UT2), MD-2 (Thursday), MD-1 (Friday), and MD (Saturday).

**Figure 2:**
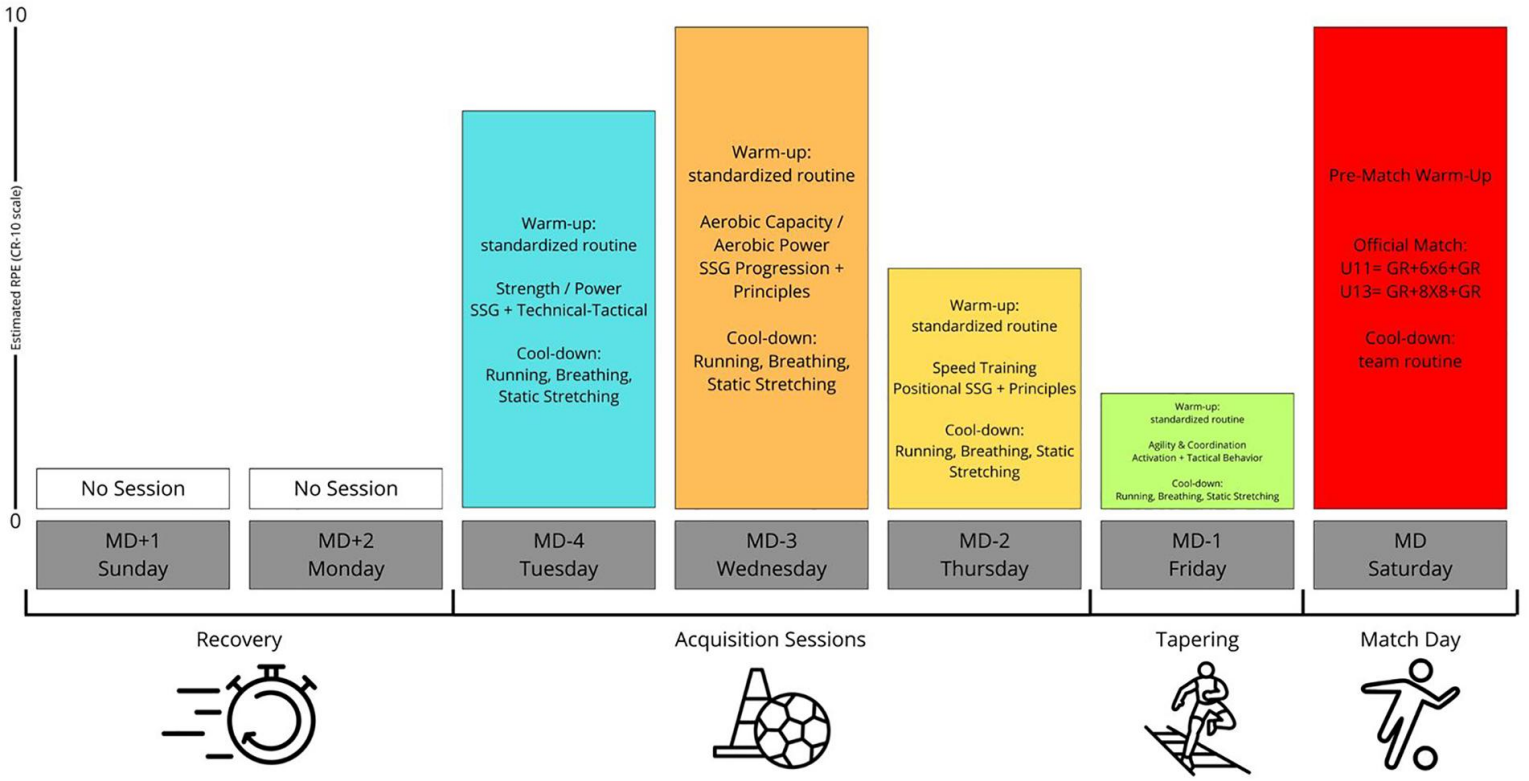
Structure of the competitive microcycle (MD-4 to MD), including training sessions and official match.

Each training session began with a standardized warm-up including low-intensity running, dynamic stretching of the main lower-limb muscle groups, technical drills, and possession-based exercises. This was followed by specific tasks emphasizing small-sided games and technical–tactical exercises, in line with the weekly plan for each age group and coordinated with the coaching staff. Matches were played under official competition rules without intervention from the investigators. U11 and U13 match consisted of two 30-minute halves, following the official regulations of the regional football association. For U11, all players were guaranteed equal playing time during the first half, as mandated by competition rules. No equivalent regulation was applied to U13 matches, where substitutions were made at the discretion of the coaching staff. Training sessions did not include mandatory rotation policies. The overall structure of the microcycle is presented in Figure 2.

### 2.4 Procedures

#### 2.4.1 External load

External load was monitored using STATSports Apex® GNSS/GPS tracking devices (Newry, Northern Ireland). The GPS units recorded raw position, velocity, and distance data at a sampling frequency of 18 Hz, and were also equipped with an accelerometer (100 Hz), a magnetometer (10 Hz), and a gyroscope (100 Hz). Each player wore the device in a pouch between the scapulae, integrated into a manufacturer-provided vest. Devices were activated 30 min prior to each session to ensure satellite signal acquisition from at least eight satellites (41). The external load variables analyzed were: total distance (m), high-speed running distance (HSR; > 18 km·h^−1^), high-intensity distance (HID; > 21 km·h^−1^), relative distance (m·min^−1^), number of sprints, maximum speed (m·s^−1^), accelerations (ACC; > 2 m·s^−2^), and decelerations (DEC; < −2 m·s^−2^). The reliability and validity of these devices are well established in the literature (42, 43), with error margins of 1%–2% in 10–18 Hz units (44). The velocity thresholds used (>18 km·h^−1^ for HSR and >21 km·h^−1^ for HID) were based on previous literature in youth football (1), but originally based on adult performance standards. These thresholds were adapted for younger players following the methodological approach described in the literature (4, 11) and are consistent with those applied in other sub-elite youth studies.

#### 2.4.2 Internal load

Objective internal load was monitored only in the U13 group using the Polar Team Pro® system (Polar Electro, Kempele, Finland), which continuously recorded heart rate during training sessions and the match. HR-derived variables were peak HR, mean HR, and the distribution of time in individualized intensity zones (Z1–Z5) expressed as %HRmax. %HRmax was defined from each player's maximal HR observed during the monitoring period. HR metrics were computed per session and summarized across the microcycle. HR monitoring was performed exclusively in the U13 group. This methodological decision was made due to both practical and physiological constraints. Specifically, chest-strap transmitters were unsuitable for U11 players because of improper fit and frequent signal loss during movement. Furthermore, heart rate in prepubertal children (10–12 years) shows high intra variability and limited reliability for estimating training intensity, as previously reported in the literature (45). For these reasons, HR monitoring was restricted to the U13 players, where physiological maturation allows for more stable and interpretable cardiac responses.

Subjective internal load was assessed using RPE Borg's CR10 scale (0–10 points) (16, 24, 46). Approximately 30 min after each training session or match, players were individually questioned by the investigator using a digital form created in Google Forms (displayed on an iPad). The scale included both numerical anchors and visual emojis to facilitate comprehension in younger athletes. Players verbally indicated their perceived exertion score, which was immediately recorded in the form. All players were already familiarized with the RPE procedure, as it had been practiced regularly for several weeks prior to data collection. SRPE was calculated by multiplying the individual RPE value by the total session duration in minutes (sRPE = RPE × duration), and expressed in arbitrary units (AU) (12, 47, 48). Data were subsequently exported and organized in Microsoft Excel® spreadsheets to ensure uniform storage and consistency.

#### 2.4.3 Recovery Status

Recovery status was assessed using the TQR scale (27), adapted to a 0–10 point version to facilitate comprehension among younger athletes (24). On this scale, 0 indicated “very poorly recovered” and 10 indicated “completely recovered.” Previous studies have integrated TQR into the monitoring of stress and perceived fatigue in youth soccer players, supporting its practical utility in this context (4, 11, 12). A familiarization period was conducted several weeks before data collection to ensure consistent responses. The TQR was administered individually, approximately 30 min before each training session, using a digital form created in Google Forms (displayed on an iPad). Players verbally indicated their recovery status while observing the adapted scale with emojis, and the score was immediately recorded. Data were subsequently exported and organized in Microsoft Excel® spreadsheets to ensure uniform storage and consistency.

#### 2.4.4 Technical-Tactical performance

Technical–tactical performance was evaluated using the FUT-SAT protocol (33) which enables the analysis of players' actions according to fundamental offensive (penetration, offensive coverage, mobility, space) and defensive (delay, defensive coverage, balance, concentration) principles during small-sided games. Two composite indicators were calculated: DMI, representing the proportion of correct decisions relative to the total number of tactical actions, and the MEI, representing the proportion of successful executions relative to total attempts. Higher DMI and MEI values indicate superior tactical awareness and execution efficiency, respectively. This approach has been validated for use in youth football populations and allows a multidimensional interpretation of performance (35).

Assessments were conducted during small-sided games (GK + 3 vs. 3 + GK) on a 36 × 27 m pitch, with each game lasting 4 min. Games were video-recorded using a GoPro HERO Action Camera (4 K, 12 MP, Wi-Fi, and Bluetooth) and subsequently analyzed using LongoMatch® software (Barcelona, Spain) (31, 49). All evaluations were performed by UEFA-licensed football coaches and sport scientists with prior experience in tactical behavior analysis. Before the observational coding, evaluators participated in a structured training and calibration session using sample video footage to ensure consistent application of the FUT-SAT criteria. Inter-rater reliability was verified during this process, yielding acceptable agreement levels across evaluators.

For each variable, ratios of successful to total actions were calculated. The action indexes were obtained based on the classification of each action as correct/incorrect for decision making or effective/ineffective for motor execution. Thus, every player action on a given principle was classified accordingly. The Decision-Making Index (DMI) quantified the proportion of correct decision-making actions relative to the total number of decision-making actions, while the Motor Effectiveness Index (MEI) quantified the proportion of effective motor executions relative to the total attempts (35). Both indexes range between 0 and 1, with higher values indicating better performance. In addition, an overall performance indicator was computed to integrate decision-making and motor effectiveness across all tactical principles. This was calculated according to Equation (1) (35):

$$\mathrm{Performance}=\frac{\sum I+\mathrm{DMI}+\mathrm{MEI}}{10},$$ (1)

where *I* represents the effectiveness index for each variable, calculated as the ratio between correct and incorrect actions (35).

#### 2.4.5 Statistical analysis

Descriptive statistics are presented as mean ± SD. External load, sRPE, and TQR were analysed using a two-way mixed ANOVA, with Session (MD-4, MD-3, MD-2, MD-1, MD) as the within-subjects factor and Age Group (U11, U13) as the between-subjects factor. The model included the main effects of Session and Age Group, as well as their interaction term (Session × Age Group). When significant main effects or interactions emerged, pairwise comparisons with Bonferroni adjustment were applied. Assumptions were checked using Shapiro–Wilk (normality), Levene's test (homogeneity), and Greenhouse–Geisser correction when sphericity was violated. Heart rate variables (U13 only) were analysed via one-way repeated-measures ANOVA across sessions (MD-4, MD-3, MD-2, MD-1, MD) with Bonferroni-adjusted pairwise comparisons. Technical–tactical differences between U11 and U13 (FUT-SAT indexes) were tested with independent-samples t-tests. Effect sizes were reported as Cohen's d for pairwise comparisons and partial eta squared (*η*²p) for ANOVA effects. Cohen's d values were interpreted as small (≈0.20), medium (≈0.50), and large (≥0.80), whereas *η*^2^*_p_* values were considered small (≈0.01), medium (≈0.06), and large (≥0.14). These thresholds allowed interpretation of both statistical and practical relevance of observed differences. Analyses were performed in IBM SPSS Statistics (IBM Corp., Armonk, NY, USA) (50, 51).

## 3 Results

### 3.1 Age-Group analysis

Table 2 presents the descriptive statistics of mean session load (training sessions and official match), recovery status and group comparisons between U11 and U13 players. Significant between-group differences were observed in most external load indicators, except for HID. Overall, U11 players exhibited greater volume and higher values for acceleration-based metrics, whereas U13 players outperformed in intensity-related measures.

**Table 2:** Mean load and recovery status comparisons by age group.

Variable	U11 (M ± SD)	U13 (M ± SD)	F	p	*η*²p	d	Post-hoc
TD (km)	4.55 ± 0.73	4.13 ± 0.83	12.92	<0.001	0.06	0.55	(a)
HSR (m)	13.57 ± 19.99	36.06 ± 29.15	39.92	<0.001	0.17	0.99	(b)
HID (m)	404.13 ± 163.11	423.70 ± 121.21	0.67	0.414	0.00	0.13	-
AvS (m/min)	49.93 ± 6.35	54.08 ± 11.27	14.39	<0.001	0.07	0.53	(b)
Sprints (n)	0.28 ± 0.63	0.70 ± 1.3	9.34	0.003	0.05	0.50	(b)
MRS (m/s)	5.98 ± 0.6	6.30 ± 0.46	12.38	<0.001	0.06	0.56	(b)
ACC (m/s²)	26.48 ± 12.85	21.52 ± 7.8	6.69	0.010	0.03	0.42	(a)
DEC (m/s²)	30.01 ± 16.63	23.26 ± 7.0	7.94	0.005	0.04	0.45	(a)
sRPE (AU)	585.20 ± 117.85	552.60 ± 96.23	5.04	0.026	0.03	0.29	(a)
TQR (AU)	7.51 ± 1.24	7.16 ± 1.56	2.60	0.108	0.01	0.26	-

Significant differences are verified as: (a) U11 > U13; (b) U13 > U11. All F values correspond to *F*(1,190).

**Figure 3:**
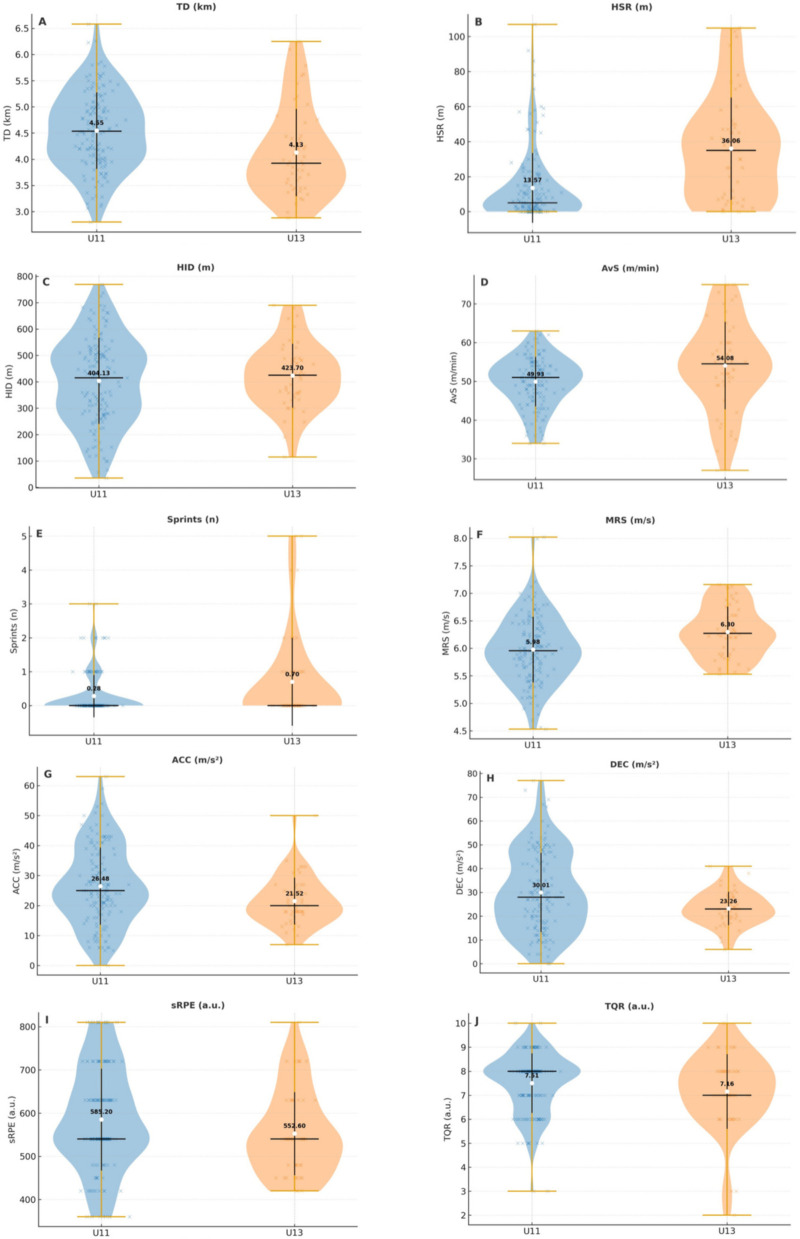
Distribution of session load and recovery status by age group. **(A–H)** external load; **(I)** internal load; **(J**) recovery status.

Between-group comparisons revealed consistent differences in external load metrics (Figure 3). U11 players covered greater total distance than U13 players [TD: *F*(1,190) = 12.92, *p* < 0.001, $\eta_{p}^{2}=0.064$; *d* = 0.55, medium], and also presented higher values for accelerations [ACC: *F*(1,190) = 6.69, *p* = 0.010, $\eta_{p}^{2}=0.034$; *d* = 0.42, small–medium] and decelerations [DEC: *F*(1,190) = 7.94, *p* = 0.005, $\eta_{p}^{2}=0.040$; *d* = 0.45, small–medium]. In contrast, U13 players outperformed U11 in intensity-related metrics, including HSR [*F*(1,190) = 39.92, *p* < 0.001, $\eta_{p}^{2}=0.174$; *d* = 0.99, large], number of sprints [*F*(1,190) = 9.34, *p* = 0.003, $\eta_{p}^{2}=0.047$; *d* = 0.50, small–medium], maximum speed [MRS: F(1,190) = 12.38, *p* < 0.001, *η*²*p* = 0.061; d = 0.56, medium], and relative distance [AvS: *F*(1,190) = 14.39, *p* < 0.001, $\eta_{p}^{2}=0.070$; *d* = 0.53, medium]. On average, U13 players reached ∼0.3 m·s^−1^ higher peak speeds and performed more than twice as many sprints per session compared to U11. No significant differences were found for HID (*p* = 0.414). For subjective measures, U11 players reported higher session-RPE values compared to U13 [*F*(1,190) = 5.04, *p* = 0.026, *η*²*p* = 0.026; *d* = 0.29, small–medium], corresponding to ∼30 AU more per session. No significant differences were observed in recovery status (TQR, *p* = 0.108).

### 3.2 Session analysis

Table 3 presents the training and match load descriptive statistics and inter sessions and match day comparisons, as well as recovery status. A consistent pattern was observed, with the match (MD) concentrating the highest intensities in specific variables, while training sessions were perceived as more demanding from a subjective perspective.

**Table 3:** Mean session load and recovery status per session.

Variable	MD-4 (M ± SD)	MD-3 (M ± SD)	MD-2 (M ± SD)	MD-1 (M ± SD)	MD (M ± SD)	F	p	η²p	d	Post-hoc
TD (km)	4.18 ± 0.68	4.42 ± 0.78	4.42 ± 0.68	4.56 ± 0.83	4.63 ± 0.85	2.39	0.053	0.05	-	-
HSR (m)	15.53 ± 20.62	22.82 ± 25.78	17.00 ± 25.02	11.15 ± 21.91	29.48 ± 26.03	4.25	0.003	0.08	0.76	(a)
HID (m)	363.40 ± 175.61	409.18 ± 138.82	416.88 ± 153.68	369.32 ± 135.60	486.35 ± 135.70	4.52	0.002	0.09	0.78–0.86	(a), (b)
AvS (m/min)	50.58 ± 8.90	50.80 ± 7.94	51.70 ± 5.84	48.08 ± 8.19	53.70 ± 8.35	3.69	0.006	0.07	0.68	(a)
Sprints (n)	0.25 ± 0.54	0.42 ± 0.96	0.33 ± 0.80	0.25 ± 0.84	0.68 ± 1.05	1.78	0.135	0.04	-	-
MRS (m/s)	5.99 ± 0.76	6.12 ± 0.50	6.03 ± 0.54	5.87 ± 0.52	6.28 ± 0.49	3.10	0.017	0.06	0.82	(a)
ACC (m/s²)	21.82 ± 12.43	23.80 ± 11.55	26.62 ± 13.82	25.98 ± 10.88	27.98 ± 10.46	1.72	0.148	0.04	-	-
DEC (m/s²)	25.70 ± 15.55	27.40 ± 15.38	29.65 ± 16.63	24.88 ± 13.13	33.98 ± 13.42	2.48	0.045	0.05	-	-
sRPE (AU)	594.00 ± 109.38	609.75 ± 102.89	630.00 ± 84.03	607.50 ± 97.21	444.00 ± 57.32	28.82	<0.001	0.38	1.72–2.59	(c)
TQR (AU)	7.25 ± 1.56	7.38 ± 1.35	7.58 ± 1.24	7.35 ± 1.33	7.55 ± 1.18	0.44	0.777	0.01	-	-

Significant differences are verified as: (a) MD > MD-1; (b) MD > MD-4; (c) Training Sessions > Match Day. All F values correspond to *F*(4,190).

**Figure 4:**
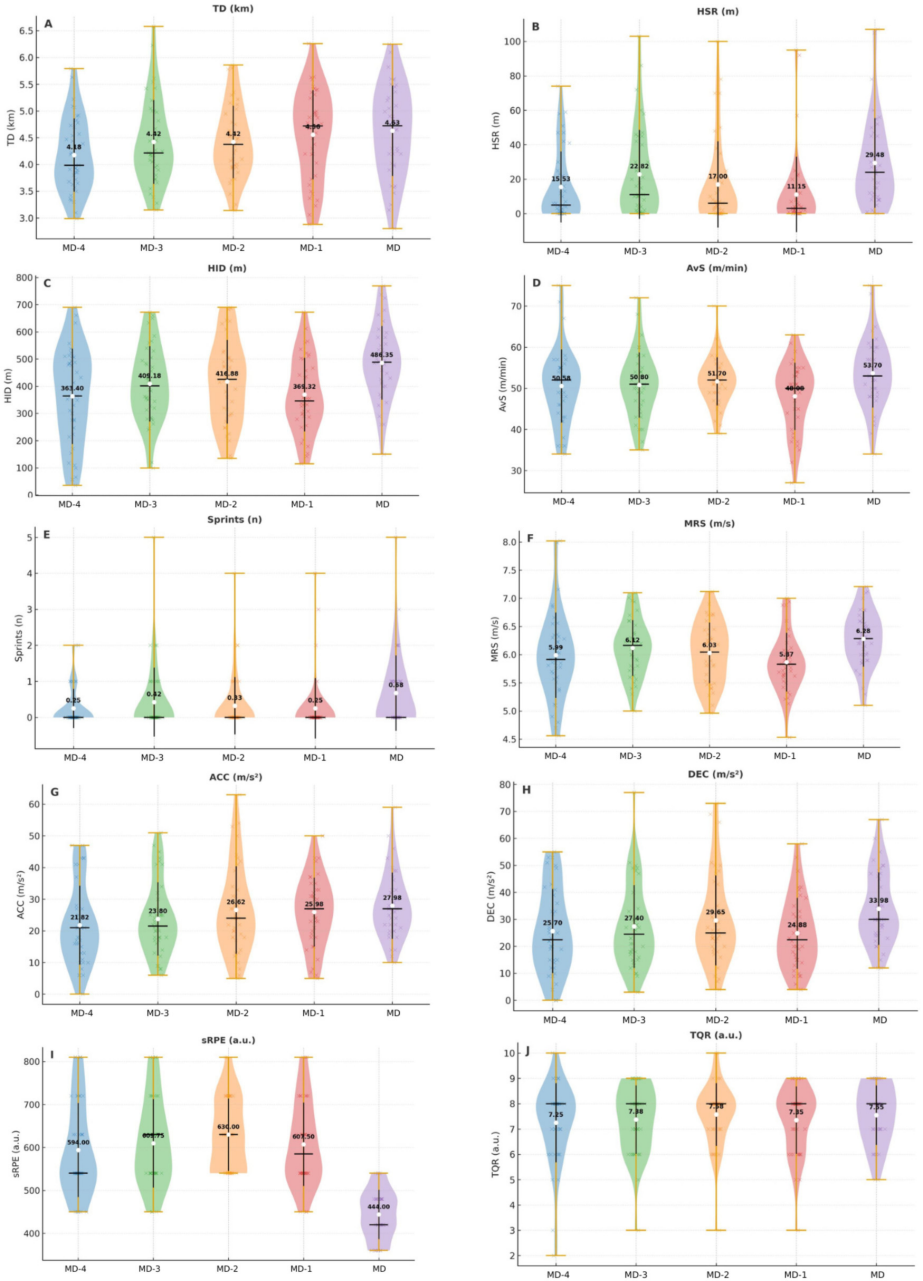
Distribution of session load and recovery status across the microcycle. **(A–H)** external load; **(I)** internal load; **(J)** recovery status.

The within-subjects analysis revealed clear differences in load distribution across the microcycle (Figure 4). For HSR, MD showed significantly higher values compared to MD-1 [*F*(4,190) = 4.25, *p* = 0.003, $\eta_{p}^{2}=0.082$; *p* = 0.007, *d* = 0.76, medium]. A similar pattern was found for MRS, where MD exceeded MD-1 [*F*(4,190) = 3.10, *p* = 0.017, $\eta_{p}^{2}=0.061$; *p* = 0.012, *d* = 0.82, large]. AvS also peaked in MD compared to MD-1 [*F*(4,190) = 3.69, *p* = 0.006, $\eta_{p}^{2}=0.072$; *p* = 0.015, *d* = 0.68, medium]. For HID, MD was superior to both MD-4 (*p* = 0.003, *d* = 0.78, medium) and MD-1 (*p* = 0.005, *d* = 0.86, large), confirming significant sessional differences [*F*(4,190) = 4.52, *p* = 0.002, $\eta_{p}^{2}=0.087$]. No significant sessional effects were found for TD, number of sprints, ACC, or DEC (all *p* > 0.05). From a subjective perspective, sRPE was markedly higher in all training sessions compared to MD [*F*(4,190) = 28.82, *p* < 0.001, $\eta_{p}^{2}=0.378$], with large magnitudes (*d* = 1.72–2.59). On average, training sessions were reported as ∼400 AU more demanding than the official match. No significant sessional differences were observed for recovery status (TQR, *p* = 0.777).

### 3.3 Session × Age group interaction analysis

Table 4 presents the descriptive statistics of mean session load (training sessions and official match) and recovery status across age groups. The Session × Age Group interaction revealed contrasting patterns in several external and internal load variables, indicating that the competitive microcycle elicited non-uniform responses between U11 and U13 players.

**Table 4:** Mean session load and recovery status per session × age group.

Variable	U11 (M ± SD)					U13 (M ± SD)					F	p	η²p	d	Post-hoc
	MD-4	MD-3	MD-2	MD-1	MD	MD-4	MD-3	MD-2	MD-1	MD					
TD (km)	4.15 ± 0.59	4.55 ± 0.78	4.58 ± 0.65	4.89 ± 0.64	4.57 ± 0.81	4.26 ± 0.95	4.04 ± 0.70	3.95 ± 0.53	3.57 ± 0.49	4.83 ± 0.95	5.99	<0.001	0.11	1.21–2.19	(a), (b), (c), (d), (e), (f), (g), (h)
HSR (m)	8.77 ± 14.53	16.60 ± 21.71	9.37 ± 18.45	9.77 ± 19.33	23.37 ± 22.17	35.80 ± 23.52	41.50 ± 29.06	39.90 ± 28.99	15.30 ± 29.16	47.80 ± 29.26	1.51	0.201	0.03	1.11–2.05	(h), (i), (j), (k), (l), (m), (n), (o), (p), (q), (r), (s), (t), (u)
HID (m)	328.33 ± 180.30	397.00 ± 149.43	415.57 ± 166.64	392.97 ± 139.55	486.80 ± 145.89	468.60 ± 111.74	445.70 ± 97.85	420.80 ± 113.32	298.40 ± 97.37	485.00 ± 105.86	2.54	0.041	0.05	0.97–1.39	(a), (v)
AvS (m/min)	47.87 ± 7.15	48.47 ± 6.57	51.00 ± 5.07	51.30 ± 5.43	51.03 ± 6.82	58.70 ± 8.96	57.80 ± 7.84	53.80 ± 7.64	38.40 ± 7.55	61.70 ± 7.60	17.01	<0.001	0.26	1.31–3.08	(a), (c), (j), (k), (m), (n), (w), (y), (z), (aa), (ab), (ac), (ad), (ae), (af), (ag), (ah)
Sprints (n)	0.17 ± 0.46	0.27 ± 0.58	0.17 ± 0.46	0.20 ± 0.66	0.60 ± 0.81	0.50 ± 0.71	0.90 ± 1.60	0.80 ± 1.32	0.40 ± 1.26	0.90 ± 1.60	0.43	0.789	0.01	-	-
MRS (m/s)	5.89 ± 0.81	6.02 ± 0.49	5.90 ± 0.52	5.85 ± 0.55	6.23 ± 0.51	6.29 ± 0.49	6.41 ± 0.43	6.43 ± 0.40	5.92 ± 0.42	6.43 ± 0.43	0.81	0.518	0.02	-	-
ACC (m/s²)	23.03 ± 13.77	25.00 ± 12.77	28.80 ± 15.12	25.67 ± 10.59	29.90 ± 11.07	18.20 ± 6.27	20.20 ± 5.83	20.10 ± 5.28	26.90 ± 12.27	22.20 ± 5.49	0.81	0.517	0.02		
DEC (m/s²)	26.17 ± 17.49	28.90 ± 17.39	32.03 ± 18.49	25.73 ± 13.55	37.20 ± 13.91	24.30 ± 7.65	22.90 ± 4.48	22.50 ± 4.53	22.30 ± 12.07	24.30 ± 4.19	0.71	0.589	0.02		
sRPE (a.u.)	624.00 ± 105.52	612.00 ± 104.10	630.00 ± 85.22	624.00 ± 97.26	436.00 ± 60.89	504.00 ± 62.93	603.00 ± 104.36	630.00 ± 84.85	558.00 ± 82.70	468.00 ± 37.95	3.45	0.010	0.07	1.13–2.88	(ai), (aj), (ak), (al), (am), (an), (ao), (ap), (aq), (ar), (as), (at), (au), (av), (aw), (ax), (ay)
TQR (a.u.)	7.37 ± 1.10	7.40 ± 1.19	7.73 ± 1.26	7.17 ± 1.42	7.87 ± 1.17	6.90 ± 2.56	7.30 ± 1.83	7.10 ± 1.10	7.90 ± 0.88	6.60 ± 0.52	2.35	0.056	0.05	-	-

Significant differences are verified as: (a) U11|MD > U13|MD-1; (b) U11|MD-1 > U11|MD-4; (c) U11|MD-3 > U13|MD-1; (d) U11|MD-2 > U13|MD-1; (e) U11|MD-1 > U13|MD-3; (f) U11|MD-1 > U13|MD-2; (g) U11|MD-1 > U13|MD-1; (h) U13|MD > U13|MD-1; (i) U13|MD > U11|MD-4; (j) U13|MD-4 > U11|MD-4; (k) U13|MD-3 > U11|MD-4; (l) U13|MD-2 > U11|MD-4; (m) U13|MD > U11|MD-3; (n) U13|MD > U11|MD-2; (o) U13|MD-4 > U11|MD-2; (p) U13|MD-3 > U11|MD-2; (q) U13|MD-2 > U11|MD-2; (r) U13|MD > U11|MD-1; (s) U13|MD-4 > U11|MD-1; (t) U13|MD-3 > U11|MD-1; (u) U13|MD-2 > U11|MD-1; (v) U11|MD > U11|MD-4; (w) U11|MD-4 > U13|MD-1; (x) U13|MD > U11|MD-3; (y) U13|MD-4 > U11|MD-3; (z) U13|MD-3 > U11|MD-3; (aa) U11|MD-3 > U13|MD-1; (ab) U13|MD > U11|MD-2; (ac) U11|MD-2 > U13|MD-1; (ad) U13|MD > U11|MD-1; (ae) U11|MD-1 > U13|MD-1; (af) U13|MD-4 > U13|MD-1; (ag) U13|MD-3 > U13|MD-1; (ah) U13|MD-2 > U13|MD-1; (ai) U11|MD-4 > U11|MD; (aj) U11|MD-3 > U11|MD; (ak) U11|MD-2 > U11|MD; (al) U11|MD-1 > U11|MD; (am) U13|MD-3 > U11|MD; (an) U13|MD-2 > U11|MD; (ao) U13|MD-1 > U11|MD; (ap) U11|MD-4 > U13|MD; (aq) U11|MD-4 > U13|MD-4; (ar) U11|MD-3 > U13|MD; (as) U11|MD-3 > U13|MD-4; (at) U11|MD-2 > U13|MD; (au) U11|MD-2 > U13|MD-4; (av) U11|MD-1 > U13|MD; (aw) U11|MD-1 > U13|MD-4; (ax) U13|MD-3 > U13|MD; (ay) U13|MD-2 > U13|MD. All F values correspond to F(4,190).

**Figure 5:**
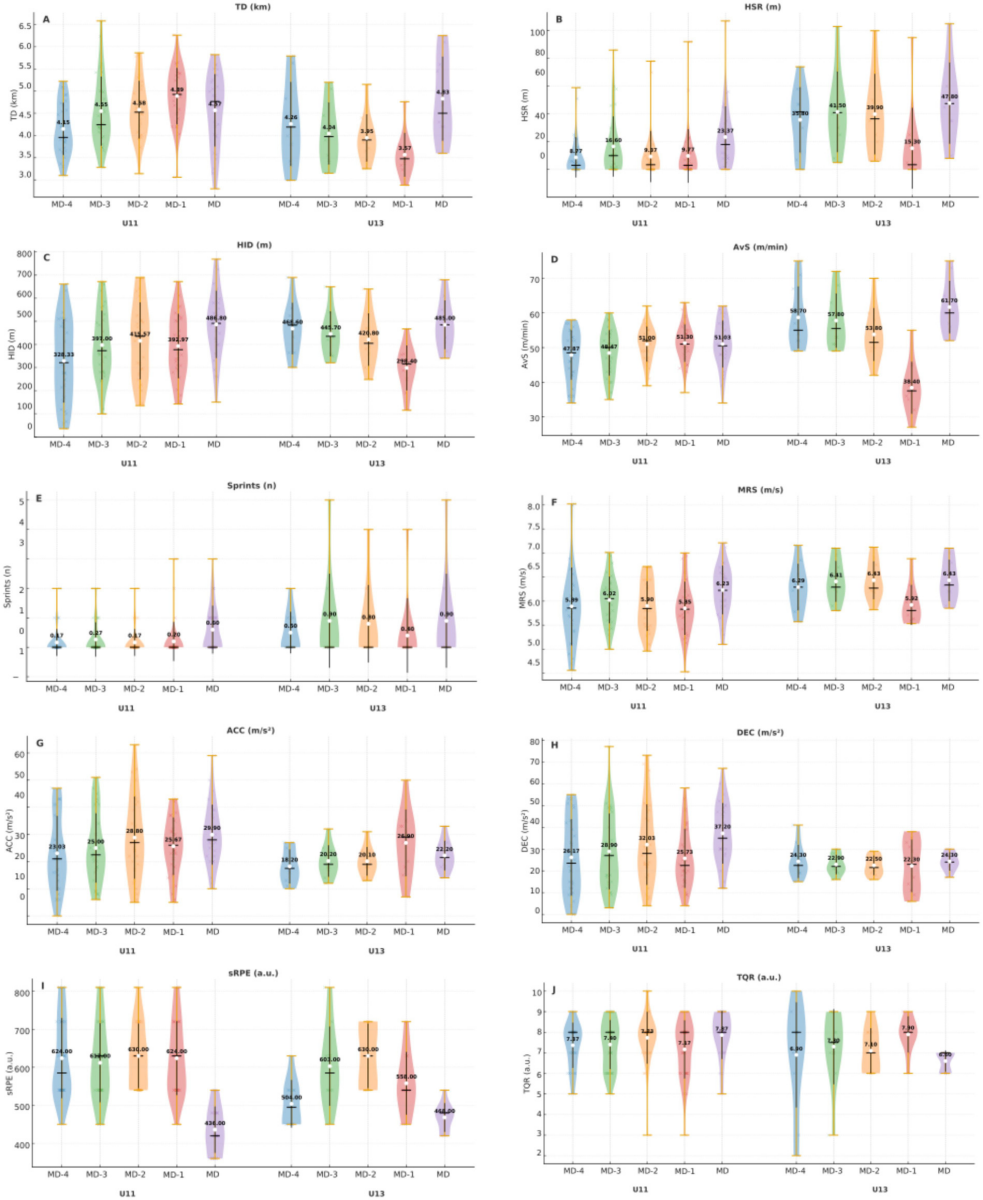
Distribution of session load and recovery status across the interaction of age group and session. **(A–H)** external load; **(I)** internal load; **(J)** recovery status.

The analysis of TD showed that U11 players in the MD covered significantly greater distances than U13 players in MD-1 [*F*(4,190) = 5.99, *p* < 0.001, $\eta_{p}^{2}=0.112$; *p* = 0.006, *d* = 1.33, large]. Within-group contrasts also revealed that U11 players covered more distance in MD-1 compared to their own MD-4 (*p* = 0.003, *d* = 1.21, large). For AvS, multiple significant contrasts emerged. U13 players displayed consistently higher values in MD compared to U11 players in MD (*p* < 0.001, *d* = 1.52, large) and showed advantages in several training sessions. However, instances favoring U11 were also observed, such as U11|MD-2 > U13|MD-1 (*p* < 0.001, *d* = 2.19, large). Regarding HID, U11 players in MD presented higher values than U11 players in MD-4 (*p* = 0.002, *d* = 0.97, large) and U13 players in MD-1 (*p* = 0.019, *d* = 1.39, large) [*F*(4,190) = 2.54, *p* = 0.041, $\eta_{p}^{2}=0.051$]. For HSR, contrasts favored U13 players in most cases. Key examples included U13|MD > U11|MD-4 (*p* < 0.001, *d* = 2.05, large), U13|MD-3 > U11|MD-4 (*p* = 0.002, *d* = 1.72, large), and U13|MD-2 > U11|MD-4 (*p* = 0.005, *d* = 1.64, large). In terms of internal load, sRPE demonstrated a robust interaction effect [*F*(4,190) = 3.45, *p* = 0.010, $\eta_{p}^{2}=0.068$]. Both age groups reported higher perceived exertion in training sessions compared to MD, but U11 consistently showed greater values than U13, with large effect sizes (*d* = 1.13–2.88). Conversely, no significant Session × Age Group interactions were identified for number of sprints, MRS, ACC, DEC, or TQR (all *p* > 0.05). These interaction patterns are summarized in Figure 5, which illustrates the distribution of session load and recovery status across age groups and sessions.

### 3.4 Heart rate analysis

Table 5 presents the mean values of HR variables and the distribution of time across intensity zones throughout the different sessions in U13 players. Overall, no significant differences were found in mean HR or maximum HR across sessions, but clear alterations emerged in the percentage of time spent in specific intensity zones.

**Table 5:** Mean values of heart rate variables across sessions in U13 players.

Variable	MD-4 (M ± SD)	MD-3 (M ± SD)	MD-2 (M ± SD)	MD-1 (M ± SD)	MD (M ± SD)	F	p	η²p	Post-hoc
Mean HR %	0.67 ± 0.02	0.66 ± 0.03	0.64 ± 0.07	0.68 ± 0.05	0.67 ± 0.01	1.23	0.312	0.10	-
Max HR %	0.88 ± 0.04	0.86 ± 0.06	0.87 ± 0.05	0.90 ± 0.03	0.90 ± 0.02	2.08	0.099	0.17	-
% Time Zone 5	0.18 ± 0.14	0.10 ± 0.04	0.07 ± 0.05	0.04 ± 0.05	0.10 ± 0.01	4.90	0.002	0.30	(a), (b)
% Time Zone 4	0.19 ± 0.08	0.19 ± 0.14	0.11 ± 0.08	0.15 ± 0.07	0.19 ± 0.01	1.99	0.113	0.15	-
% Time Zone 3	0.18 ± 0.07	0.20 ± 0.09	0.20 ± 0.07	0.24 ± 0.08	0.23 ± 0.02	1.01	0.414	0.08	-
% Time Zone 2	0.14 ± 0.04	0.18 ± 0.07	0.22 ± 0.10	0.27 ± 0.05	0.18 ± 0.02	6.79	<0.001	0.38	(c), (d), (e)
% Time Zone 1	0.31 ± 0.07	0.33 ± 0.15	0.43 ± 0.16	0.30 ± 0.14	0.29 ± 0.02	2.01	0.110	0.15	-

Significant differences are verified as: (a) MD-4 > MD-2; (b) MD-4 > MD-1; (c) MD-1 > MD; (d) MD-1 > MD-4; (e) MD-1 > MD-3. All F values correspond to F(4,450).

**Figure 6:**
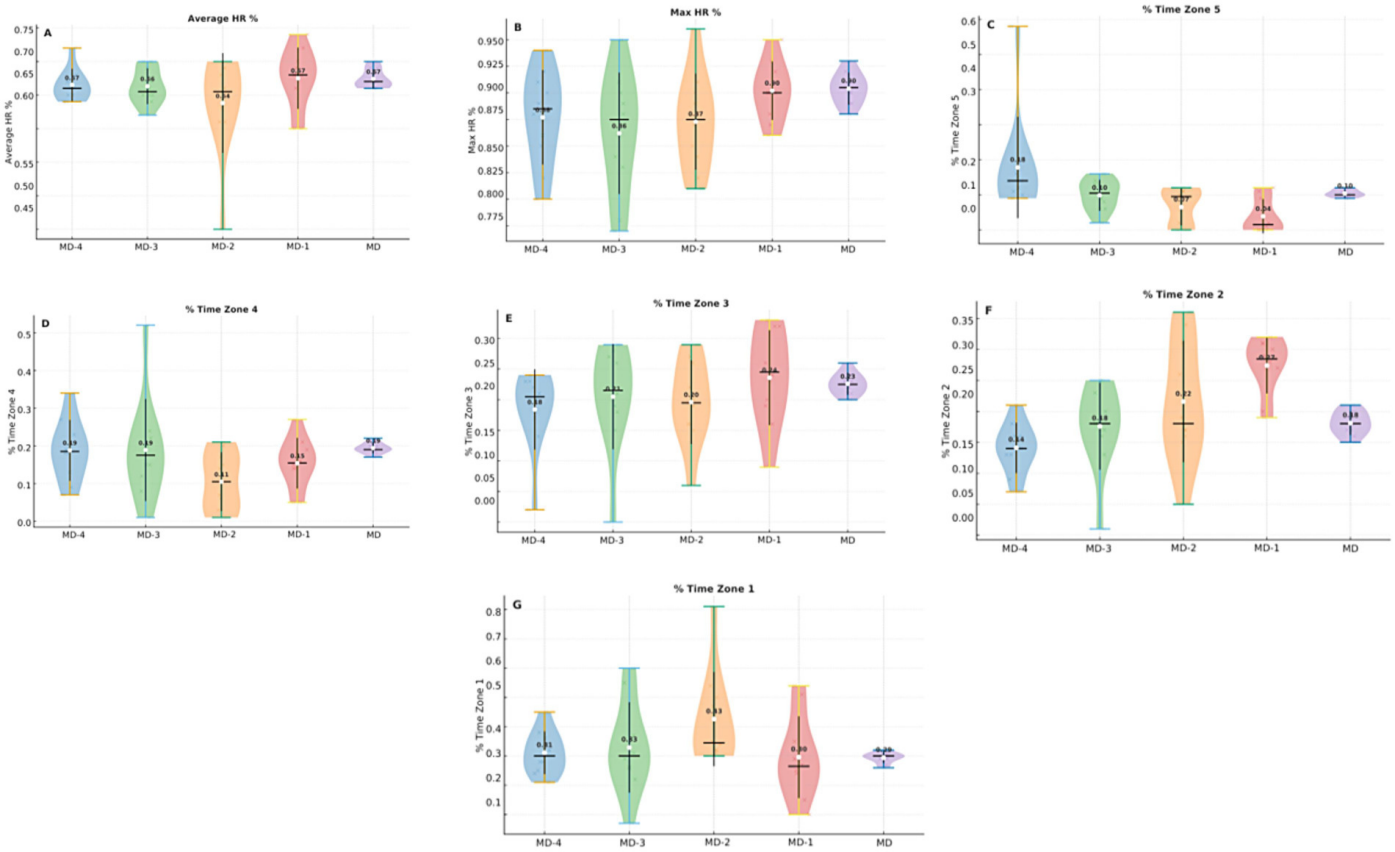
Distribution of HR variables across sessions for U13 players. **(A,B)** average and maximum HR; **(C–G)** time spent in intensity zones.

Figure 6 complements these results by displaying the intra dispersion of HR responses, highlighting the variability between players. This graphical representation illustrates that, although average values were relatively stable across sessions, there was substantial heterogeneity in the time spent at both the highest (Z5) and lowest (Z2) intensity zones.

### 3.5 Technical-Tactical analysis

Detailed principle-level data are presented in Tables 6–8 for transparency. No significant differences were found in the frequency of actions or in most offensive and defensive principles, whether expressed in absolute values, relative effectiveness, decision-making, or motor efficiency (all *p* > 0.05). These findings suggest broadly similar technical–tactical profiles between the two age groups. However, offensive coverage consistently emerged as the only principle with significant differences, with U13 players achieving higher effectiveness in both decision-making and motor execution (*p* < 0.01; *d* ≈ 0.7, medium).

**Table 6:** Technical–tactical performance (FUT-SAT) outcomes per age group.

Variable	U11 (M ± SD)	U13 (M ± SD)	t(df)	p	d	Post-hoc
**Frequency (n)**						
Actions	13.37 ± 2.25	13.80 ± 2.20	*t* = −0.54 (df≈16)	0.599	0.19	–
Errors	3.37 ± 1.75	3.90 ± 1.20	*t* = −1.08 (df≈23)	0.293	0.33	–
Opposite Field Actions	6.70 ± 1.88	6.00 ± 1.15	*t* = 1.40 (df≈26)	0.174	0.40	–
Penetration	2.73 ± 1.05	2.80 ± 1.03	*t* = −0.18 (df≈16)	0.862	0.06	–
Offensive Coverage	1.43 ± 1.17	1.60 ± 0.84	*t* = −0.49 (df≈21)	0.630	0.15	–
Mobility	1.67 ± 0.96	1.70 ± 0.82	*t* = −0.11 (df≈18)	0.917	0.04	–
Space	1.27 ± 0.94	1.80 ± 0.79	*t* = −1.76 (df≈18)	0.095	0.59	–
Delay	2.37 ± 1.25	2.30 ± 1.06	*t* = 0.16 (df≈18)	0.871	0.06	–
Defensive Coverage	1.40 ± 0.81	0.90 ± 0.74	*t* = 1.81 (df≈17)	0.088	0.63	–
Balance	1.40 ± 1.16	1.70 ± 1.34	*t* = −0.63 (df≈14)	0.536	0.25	–
Concentration	1.10 ± 0.84	0.90 ± 0.88	*t* = 0.63 (df≈15)	0.538	0.23	–
**Effectiveness (%)**						
Sucess	0.74 ± 0.14	0.71 ± 0.10	*t* = 0.59 (df≈23)	0.560	0.18	–
Errors	0.26 ± 0.14	0.29 ± 0.10	*t* = −0.59 (df≈23)	0.560	0.18	–
Penetration	0.75 ± 0.26	0.65 ± 0.21	*t* = 1.17 (df≈19)	0.257	0.38	–
Offensive Coverage	0.64 ± 0.45	0.95 ± 0.16	*t* = −3.17 (df≈38)	0.003	0.76	(a)
Mobility	0.64 ± 0.41	0.72 ± 0.34	*t* = −0.57 (df≈18)	0.575	0.19	–
Space	0.66 ± 0.44	0.78 ± 0.37	*t* = −0.86 (df≈18)	0.399	0.29	–
Delay	0.74 ± 0.28	0.62 ± 0.40	*t* = 0.88 (df≈12)	0.398	0.39	–
Defensive Coverage	0.68 ± 0.43	0.50 ± 0.47	*t* = 1.06 (df≈14)	0.309	0.40	–
Balance	0.68 ± 0.42	0.70 ± 0.42	*t* = −0.11 (df≈15)	0.915	0.04	–
Concentration	0.43 ± 0.47	0.40 ± 0.46	*t* = 0.16 (df≈16)	0.871	0.06	–

Significant differences are verified as: (a): U13 > U11.

**Table 7:** Absolute values of decision making and motor efficiency per game principle.

Variable	U11 (M ± SD)	U13 (M ± SD)	t(df)	p	d	Post-hoc
**Decision Making for each game principle**						
Penetration	2.10 ± 1.03	1.80 ± 0.79	*t* = 0.96 (df≈20)	0.348	0.31	–
Offensive Coverage	1.30 ± 1.15	1.50 ± 0.85	*t* = −0.59 (df≈21)	0.564	0.18	–
Mobility	1.33 ± 0.84	1.20 ± 0.79	*t* = 0.45 (df≈16)	0.655	0.16	–
Space	1.07 ± 0.69	1.30 ± 0.82	t = −0.81 (df≈13)	0.434	0.32	–
Delay	1.90 ± 1.12	1.40 ± 0.97	*t* = 1.36 (df≈18)	0.191	0.46	–
Defensive Coverage	1.13 ± 0.82	0.70 ± 0.67	*t* = 1.66 (df≈19)	0.113	0.55	–
Balance	1.03 ± 0.85	1.30 ± 1.16	*t* = −0.67 (df≈12)	0.515	0.29	–
Concentration	0.63 ± 0.76	0.60 ± 0.70	*t* = 0.13 (df≈17)	0.900	0.04	–
**Motor Efficiency for each game principle**						
Penetration	1.87 ± 1.01	1.70 ± 0.82	*t* = 0.52 (df≈19)	0.607	0.17	–
Offensive Coverage	1.20 ± 1.16	1.30 ± 0.48	*t* = −0.38 (df≈36)	0.703	0.10	–
Mobility	1.10 ± 0.84	1.00 ± 0.67	*t* = 0.38 (df≈19)	0.706	0.12	–
Space	0.97 ± 0.72	1.00 ± 0.67	*t* = −0.13 (df≈17)	0.895	0.05	–
Delay	1.30 ± 0.88	1.20 ± 0.92	*t* = 0.30 (df≈15)	0.767	0.11	–
Defensive Coverage	0.87 ± 0.73	0.60 ± 0.52	*t* = 1.26 (df≈22)	0.219	0.39	–
Balance	0.83 ± 0.83	1.20 ± 0.92	*t* = −1.12 (df≈14)	0.282	0.43	–
Concentration	0.53 ± 0.68	0.60 ± 0.70	*t* = −0.26 (df≈15)	0.796	0.10	–

**Table 8:** Decision-Making Index (DMI), motor efficiency Index (MEI), and overall performance per game principle.

Variable	U11 (M ± SD)	U13 (M ± SD)	t(df)	p	d	Post-hoc
**Decision Making Index**						
DMI Index	0.77 ± 0.13	0.70 ± 0.12	*t* = 1.54 (df≈18)	0.142	0.52	–
Penetration	0.77 ± 0.24	0.65 ± 0.21	*t* = 1.55 (df≈18)	0.140	0.52	–
Offensive Coverage	0.66 ± 0.46	0.95 ± 0.16	*t* = −3.03 (df≈38)	0.004	0.72	(a)
Mobility	0.69 ± 0.40	0.62 ± 0.39	*t* = 0.52 (df≈16)	0.609	0.19	–
Space	0.73 ± 0.41	0.78 ± 0.37	*t* = −0.40 (df≈17)	0.693	0.14	–
Delay	0.83 ± 0.23	0.62 ± 0.40	*t* = 1.62 (df≈11)	0.133	0.77	–
Defensive Coverage	1.13 ± 0.82	0.70 ± 0.67	*t* = 1.66 (df≈19)	0.113	0.55	–
Balance	1.03 ± 0.85	1.30 ± 1.16	*t* = −0.67 (df≈12)	0.515	0.29	–
Concentration	0.63 ± 0.76	0.60 ± 0.70	*t* = 0.13 (df≈17)	0.900	0.04	–
**Motor Efficiency Index**						
MEI Index	0.64 ± 0.15	0.62 ± 0.10	*t* = 0.49 (df≈25)	0.630	0.14	–
Penetration	0.70 ± 0.28	0.60 ± 0.17	*t* = 1.35 (df≈25)	0.188	0.39	–
Offensive Coverage	0.58 ± 0.47	0.88 ± 0.19	*t* = −2.91 (df≈36)	0.006	0.73	(a)
Mobility	0.57 ± 0.40	0.53 ± 0.38	*t* = 0.30 (df≈17)	0.769	0.10	–
Space	0.67 ± 0.42	0.60 ± 0.40	*t* = 0.45 (df≈16)	0.662	0.16	–
Delay	0.60 ± 0.32	0.55 ± 0.42	*t* = 0.33 (df≈13)	0.748	0.14	–
Defensive Coverage	0.55 ± 0.44	0.50 ± 0.47	*t* = 0.29 (df≈15)	0.772	0.11	–
Balance	0.50 ± 0.45	0.68 ± 0.41	*t* = −1.16 (df≈17)	0.262	0.40	–
Concentration	0.39 ± 0.47	0.40 ± 0.46	*t* = −0.07 (df≈16)	0.948	0.02	–
**Overall Perfomance**						
Performance	0.66 ± 0.17	0.66 ± 0.10	*t* = −0.02 (df≈27)	0.980	0.01	

Significant differences are verified as: (a): U13 > U11.

**Figure 7:**
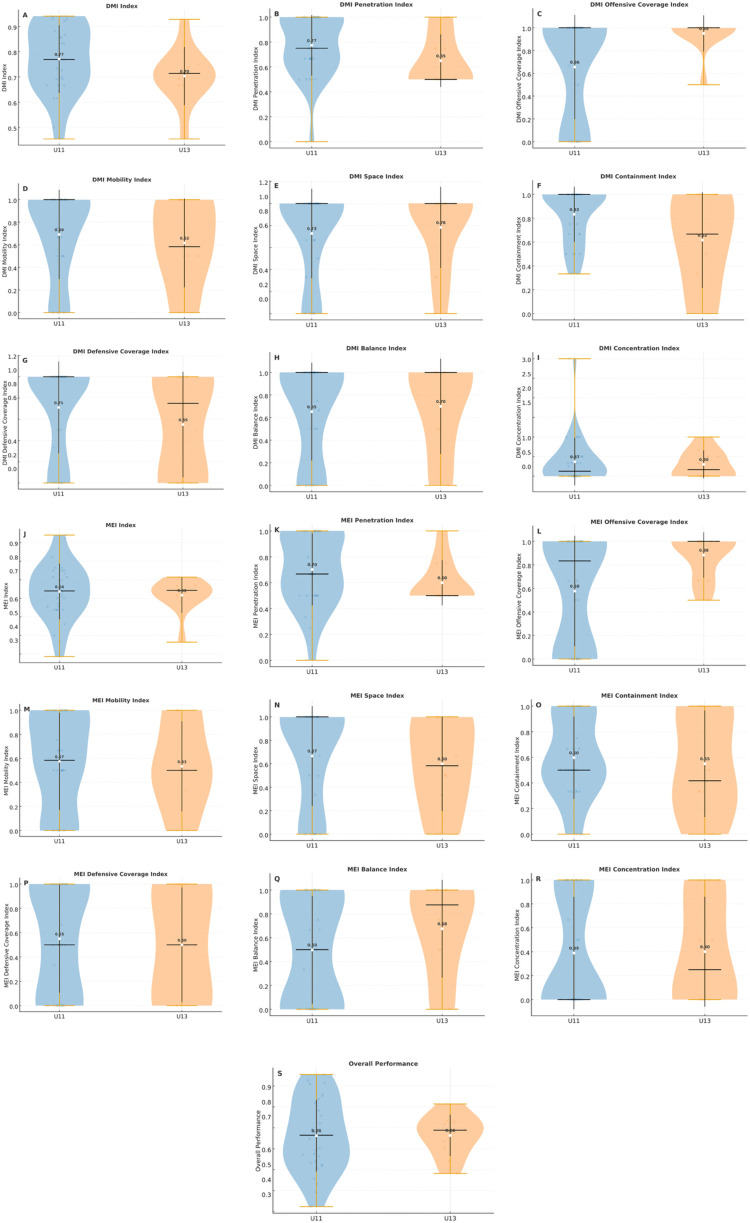
Distribution of DMI and MEI Index values per game principle and age group. (**A–I)** DMI 410 per principle; (**J–R)** MEI per principle; S: overall performance.

Figure 7 complements these results by illustrating the distribution of DMI and MEI values across game principles for each age group. The graphical representation highlights the superior performance of U13 players in offensive coverage, while confirming the absence of meaningful differences between groups in the remaining principles and in overall performance.

## 4 Discussion

The present study aimed to analyze intra and inter variation of external load, internal load, recovery, and technical–tactical indicators across a competitive microcycle in sub-elite U11 and U13 football players. These two age groups were intentionally selected because they represent a crucial developmental transition, from late childhood to early adolescence, times when perceptual, physical, and tactical abilities start to differentiate more clearly. Understanding these differences is important for coaches and practitioners, as it helps determine how training stimuli should be scaled, how recovery should be managed, and how tactical instruction can be progressively structured to match developmental readiness. The hypotheses were: (i) the match would elicit the highest objective intensities, whereas training sessions would be perceived as more demanding; and (ii) U13 players would outperform U11 in high-intensity and technical–tactical outcomes, while U11 players would show higher perceived exertion and greater motor irregularity.

Regarding the first hypothesis, our findings confirmed that the MD concentrated the highest intensities in specific external-load variables. Across sessions, MD exceeded MD-1 in HSR, MRS and AvS, and surpassed both MD-4 and MD-1 in HID. In contrast, sRPE was markedly higher in all training sessions than in MD, averaging approximately 400 AU more. This difference likely reflects not only greater cumulative psychophysiological strain but also the longer duration of training sessions compared with the match. Because sRPE is calculated as the product of RPE and session duration, elevated values may result from higher perceived effort, extended session length, or both. This dual interpretation underscores the importance of considering RPE and sRPE jointly when evaluating internal load, particularly in youth players whose perceptual responses may be influenced by session structure and contextual demands. Still, these patterns are consistent with a structured microcycle culminating in competition as the most intense stimulus, with a pre-match taper (e.g., lower MD-1 intensity) reflected in our MD-1 results. The second hypothesis was only partially supported. Between groups, U13 players consistently outperformed U11 in intensity- and velocity-related variables (HSR, number of sprints, MRS, AvS), whereas U11 covered more TD and performed more ACC/DEC and reported higher sRPE. Notably, no between-group difference emerged for HID, reinforcing that age-related contrasts were concentrated on velocity-/intensity-oriented indicators and movement regulation, rather than on total HID *per se*. Taken together, this profile suggests greater motor efficiency and capacity to reach high intensities in U13, and greater volume with more frequent speed changes (ACC/DEC) and higher perceived strain in U11.

At the within-subject level (sessions), the microcycle displayed clear distribution effects: MD > MD-1 in HSR, MRS and AvS (and MD > MD-4/MD-1 in HID), while TD, sprints, ACC and DEC did not vary significantly by session. From a subjective standpoint, training sessions were consistently more demanding than the match (all training sessions > MD in sRPE), and TQR remained stable across the week (no sessional differences). These results reinforce the practical separation between objective intensity (peaking in MD) and perceived demand (peaking during training), with recovery perceptions not fluctuating meaningfully at the session level.

Session × age-group interactions clarified how these dynamics differed between U11 and U13. Significant interactions were observed for TD, AvS, HID and sRPE, indicating non-uniform responses across the microcycle. For example, U13 displayed consistently higher AvS in MD than U11, and multiple cross-session contrasts favored U13 for HSR (e.g., U13|MD > U11|MD-4), while U11 showed advantages in specific TD contrasts (e.g., U11|MD-1 > U11|MD-4). Importantly, no interactions emerged for sprints, MRS, ACC, DEC or TQR, aligning with the age-group main-effect pattern (velocity/intensity vs. volume/ACC-DEC) and with the sessional stability of TQR.

Heart rate responses were only analysed in U13, which limited cross-age comparisons. Still, the distribution across intensity zones showed meaningful variations: higher time in Z5 at the start of the week and greater Z2 exposure on MD-1, supporting the existence of programmed intensity fluctuations. Although these findings are consistent with load tapering strategies, the lack of HR data in U11—due to practical issues with chest straps and the variability of prepubertal HR responses (45) represents a key limitation and should be addressed in future research. Future studies should integrate age-appropriate HR technologies to enable direct cross-age comparisons of internal physiological load (3, 11).

In the technical–tactical domain, inter-group differences were concentrated in offensive coverage, where U13 players demonstrated higher effectiveness in both DMI and MEI. This principle reflects the player's ability to provide effective support to the ball carrier, ensuring passing options and team continuity during possession. Such behavior demands not only spatial awareness and game reading, but also anticipatory coordination and communication with teammates—skills that typically consolidate between ages 11 and 13, when perceptual–cognitive and motor capacities become more integrated. Although the FUT-SAT protocol has been validated in youth football, it was originally designed for players aged 13 years and older. Therefore, its application in prepubertal players (U11) should be interpreted with caution, as perceptual–cognitive and motor processes may not yet be fully consolidated at this stage. These developmental constraints might partially influence tactical consistency and decision-making precision in younger players, potentially affecting comparisons between age groups. Nonetheless, the protocol remains a valuable framework for identifying early patterns of tactical understanding and motor execution in formative stages.

These findings align with previous evidence showing that older youth players exhibit superior tactical cooperation and spatial-temporal adjustment (33, 52, 53). Specifically, one study (54) described offensive coverage as a principle that evolves with increased understanding of collective play, whereas younger players often focus on the ball rather than off-the-ball positioning. Consequently, U13 players' greater success in this principle may indicate a developmental transition from egocentric to allocentric tactical processing, enabling better synchronization within team play. When contrasted with the literature—which examined decision-making during a single 3v3 SSG—our weekly monitoring indicates that cumulative exposure across training and match contexts amplifies age-related differences, particularly in offensive coverage. The same study (35) reported moderate DMI/MEI values and position-specific effects (e.g., contention), our data across a full microcycle revealed a marked superiority of U13 in offensive coverage, suggesting that principle-specific expertise may become more evident when assessed over multiple sessions rather than in an isolated SSG. Methodological differences (isolated task vs. microcycle, positional constraints, accumulated fatigue/learning) plausibly explain why distinct principles discriminate in each study (contention vs. offensive coverage), yet both converge in showing that tactical effectiveness is principle-specific and development-sensitive.

Overall, these results emphasize the value of a multidimensional monitoring approach in youth football. Combining external load (TD, AvS, HSR, sprints, ACC/DEC), internal load (sRPE; HR), recovery (TQR), and technical–tactical performance (FUT-SAT: DMI, MEI) provides a granular view of weekly demands and developmental differences. Practically, coaches should: (i) plan training to manage the higher perceived cost of sessions relative to matches; (ii) target motor efficiency in U11 (reducing unnecessary ACC/DEC while sustaining quality), and high-intensity capacity and offensive support behaviors in U13; and (iii) consider principle-specific tactical development with repeated exposure across the week. Future research should prioritize extending HR monitoring to younger age groups using age-appropriate technologies to overcome the limitations of chest-strap methods noted in prepubertal cohorts. In parallel, combining microcycle-level monitoring with standardized small-sided game protocols would allow researchers to disentangle the acute effects of specific tasks from the cumulative impact of training and match exposure on tactical behavior. Moreover, future studies should integrate biological maturation markers, as growth and maturity status are likely to influence the relationships between physical, perceptual, and tactical domains. From a long-term perspective, the age-related contrasts observed here have direct implications for athlete development planning. The higher motor irregularity and perceived strain in U11 players suggest that early-stage training should prioritize motor control, efficiency, and technical adaptability before progressively emphasizing intensity and tactical synchronization. Conversely, U13 players' improved efficiency and offensive coordination indicate readiness for more complex, game-oriented and decision-based tasks. Aligning training progression with these developmental profiles may optimize both performance and learning trajectories in sub-elite youth football. Finally, longitudinal approaches linking training load, perceptual indicators, and GPAI/FUT-SAT outcomes could clarify how repeated weekly exposures contribute to the long-term development of sport-specific fundamental motor skills, ensuring that physical, perceptual, and tactical progressions are aligned throughout the formative years of sub-elite football.

### 4.1 Practical applications

This study provides preliminary insights into training design and monitoring in sub-elite youth football. Matches elicited the highest intensities in objective metrics, while training sessions were perceived as more demanding at the psychophysiological level, supporting the use of sRPE alongside external load data. Age-related differences also suggest that training should be tailored to developmental stage: U13 players showed greater tolerance to high-intensity efforts, whereas U11 players displayed higher motor irregularity (ACC/DEC, total distance) and greater perceived exertion. From a technical–tactical perspective, the superiority of U13 players in offensive coverage highlights the importance of integrating decision-making and coordination tasks at early stages.

Practically, these findings provide clear guidance for coaches and practitioners. Training loads and session structures should be adjusted to reflect each group's physiological and perceptual characteristics. For U11 players, sessions should prioritize motor efficiency, stability in movement patterns, and control of accelerations/decelerations—using shorter and more varied formats that encourage correct execution without excessive fatigue. For U13 players, training can include higher-intensity drills that combine physical and tactical elements, such as position-specific small-sided games designed to replicate match demands and promote communication and anticipation. Coaches can use sRPE to monitor perceived exertion in real time, TQR to gauge recovery status before sessions, and FUT-SAT periodically to assess how tactical understanding evolves with cumulative load exposure across the week. Integrating these tools allows for a dynamic feedback loop between training prescription, player response, and adaptation—optimizing long-term skill development and reducing the risk of overload.

Several limitations must be acknowledged. The sample was relatively small and imbalanced between age groups (U11 *n* = 30; U13 *n* = 10), which may limit the generalizability of results and increase the influence of individual variability, particularly in the older group. Heart-rate data were collected only in U13 players, restricting cross-age comparisons and limiting conclusions about the development of internal physiological responses. No measures of biological maturation or growth status were included, which may have influenced inter-individual variability. The sample was also limited to a single sub-elite academy, reducing generalizability. Future research should integrate HR across ages, include maturation markers, and apply multi-method approaches to assess tactical performance in both training and competition.

Taken together, these findings reinforce the value of a multidimensional monitoring framework that integrates external load, internal load, recovery, and technical–tactical behavior. While each variable provides relevant insights in isolation, their combined interpretation offers a clearer picture of how training stimuli interact during player development.

## 5 Conclusions

In sub-elite youth football, the match represented the highest-intensity stimulus in objective metrics, while training sessions elicited higher perceived exertion. Between groups, U13 players outperformed U11 in high-speed and velocity-based measures, whereas U11 players showed higher total distance, accelerations, decelerations, and sRPE, indicating lower motor efficiency and greater subjective effort. Technical–tactical differences were limited, with U13 players displaying superior effectiveness in offensive coverage, as reflected in both DMI and MEI indexes.

## Data Availability

The raw data supporting the conclusions of this article will be made available by the authors, without undue reservation.
